# Routine kV‐CBCT quality assurance in IGRT: Workflow‐based comparison of QUART/MaximQA vs Catphan/ARTISCAN

**DOI:** 10.1002/acm2.70708

**Published:** 2026-07-15

**Authors:** Karolina Sanocka, Bartosz Pawałowski, Ewelina Nowak, Zuzanna Wróblewicz, Urszula Sobocka‐Kurdyk, Olga Jankowiak, Krzysztof Matuszewski, Maksymilian Wosicki, Sebastian Gajny, Tomasz Piotrowski

**Affiliations:** ^1^ Department of Medical Physics Greater Poland Cancer Centre Poznan Poland; ^2^ Faculty of Health Sciences Calisia University Kalisz Poland; ^3^ Department of Electroradiology Poznan University of Medical Sciences Poznan Poland

**Keywords:** ARTISCAN, Catphan, image quality, kV‐CBCT, MaximQA, QA phantom, QUART

## Abstract

**Purpose:**

The study aimed to compare image‐quality metrics measured by two kV‐CBCT QA systems—the QUART phantom with MaximQA and the Catphan phantom with ARTISCAN—using standard clinical kV‐CBCT imaging protocols in a Varian TrueBeam IGRT setting, in line with AAPM TG‐142/TG‐179 QA recommendations.

**Methods:**

QA scans of the QUART and Catphan phantoms were acquired for image gently, head, thorax, pelvis and pelvis large imaging protocols. Key image quality metrics (noise, signal‐to‐noise ratio [SNR], uniformity, geometric distortion, Hounsfield unit [HU] constancy, contrast‐to‐noise ratio [CNR]) were measured using MaximQA and ARTISCAN software. Descriptive statistics and paired t‐tests (α = 0.01) evaluated differences between systems; TG‐142/TG‐179 tolerances provided context.

**Results:**

The QUART/MaximQA system consistently produced significantly lower measured image noise and higher measured SNR than Catphan/ARTISCAN across most protocols (e.g., Pelvis Large: SNR 495.1 ± 40.7 vs 283.6 ± 31.5, *p* < 0.01). Geometric accuracy was excellent in both systems (measured distances ∼99.6%–100% of nominal, no significant differences). QUART phantom HU values were closer to nominal for inserts; however, the Catphan Solid Water insert was interpreted in terms of material‐related HU behavior and stability rather than poorer HU accuracy (e.g., water‐equivalent insert: QUART ≈ 0 HU vs +60 for Catphan/Solid Water, *p* < 0.01). Uniformity differences were small (< 5 HU) and often not significant. CNR and low‐contrast detectability were reported as complementary QA indicators rather than directly equivalent metrics. Overall, both systems yielded image quality metrics within TG‐142/TG‐179 baselines.

**Conclusions:**

Both QUART/MaximQA and Catphan/ARTISCAN provide consistent kV‐CBCT QA metrics, but due to differences in phantom design, ROI definition, analysis software and setup conditions, their absolute values should not be considered directly interchangeable. The choice of phantom/software may influence absolute QA values. We recommend using institution‐specific baselines per TG‐142/TG‐179 and cite emerging literature on automated kV‐CBCT QA.

## 1 INTRODUCTION

kV‐CBCT imaging plays a crucial role in image‐guided radiation therapy (IGRT) for patient setup and adaptation. The quality of these images directly impacts localization accuracy and treatment outcomes, making regular quality assurance (QA) essential. Common QA phantoms used in clinics include, for example, the QUART DVT_VN_6/3/6 phantom (QUART GmbH, Zorneding, Germany), which may be utilized with software such as MaximQA (Varian Medical Systems, a Siemens Healthineers company, Palo Alto, CA, USA), as well as the Catphan 604 phantom (The Phantom Laboratory, Salem, NY, USA), which can be analyzed using tools such as ARTISCAN (AQUILAB, Loos‐les‐Lille, France). While both phantoms are regularly employed, their comparative performance has not been systematically evaluated. As a result, users may rely on vendor tolerances or single QA methods, leading them to overlook discrepancies between systems.^1^, ^2^, ^3^, ^4^ AAPM Task Group 179 provides guidelines for QA in CT‐based IGRT, emphasizing the importance of checking imaging geometry, contrast resolution, noise, uniformity, and dose consistency. However, neither the QUART/MaximQA nor the Catphan/ARTISCAN tools have a universally established baseline defined in TG‐179. This lack of a defined standard creates uncertainty regarding how well these tools align with one another and with clinical tolerances.^3^

This study aims to critically compare the QUART/MaximQA and Catphan/ARTISCAN systems for routine kV‐CBCT QA. Although both methods can detect temporal trends in image quality, they may produce different absolute values due to variations in their phantom designs. Understanding these differences will help practitioners select or combine QA tools more effectively. We will analyze key metrics such as signal‐to‐noise ratio (SNR), contrast‐to‐noise ratio (CNR), Hounsfield unit (HU) uniformity, spatial resolution, and geometric accuracy for identical CBCT protocols, and assess the statistical agreement between the two QA approaches.

## 2 MATERIAL AND METHODS

### 2.1 Equipment and imaging protocols

QA measurements were performed on a Varian TrueBeam linear accelerator equipped with the HyperSight, the newest kV‐CBCT system (Varian Medical Systems, a Siemens Healthineers company, Palo Alto, CA, USA). Calibration and warm‐up were done per vendor routine before each session. The QUART phantom was positioned on the treatment couch according to the routine MaximQA procedure, whereas the Catphan phantom was mounted on its dedicated holder with reduced couch contribution. Thus, both datasets used the same clinical kV‐CBCT protocols and reconstruction settings, but not fully identical physical setup conditions; this was considered when interpreting absolute image‐quality values. Scans were performed according to clinical IGRT protocols (Table 1).^5^

**TABLE 1:** Key acquisition parameters of the kV‐CBCT protocols used in the analysis.

Protocol	kV	mAs per projection [mAs/frame]	Acquisition time [s]	Scan trajectory	Fan type
Image Gently	80	0.20	22.2	Half	Full‐fan
Head	100	0.30	22.2	Half	Full‐fan
Thorax	125	0.30	40.0	Full	Half‐fan
Pelvis	125	1.20	40.0	Full	Half‐fan
Pelvis Large^*^	140	1.88	40.0	Full	Half‐fan

*For Pelvis Large, the mAs/frame value results from the exposure settings (75 mA, 25 ms), that is, 1.875 mAs/projection; the table may report the rounded value 1.88.^5^

### 2.2 Phantom and software

#### 2.2.1 The QUART phantom

**FIGURE 1:**
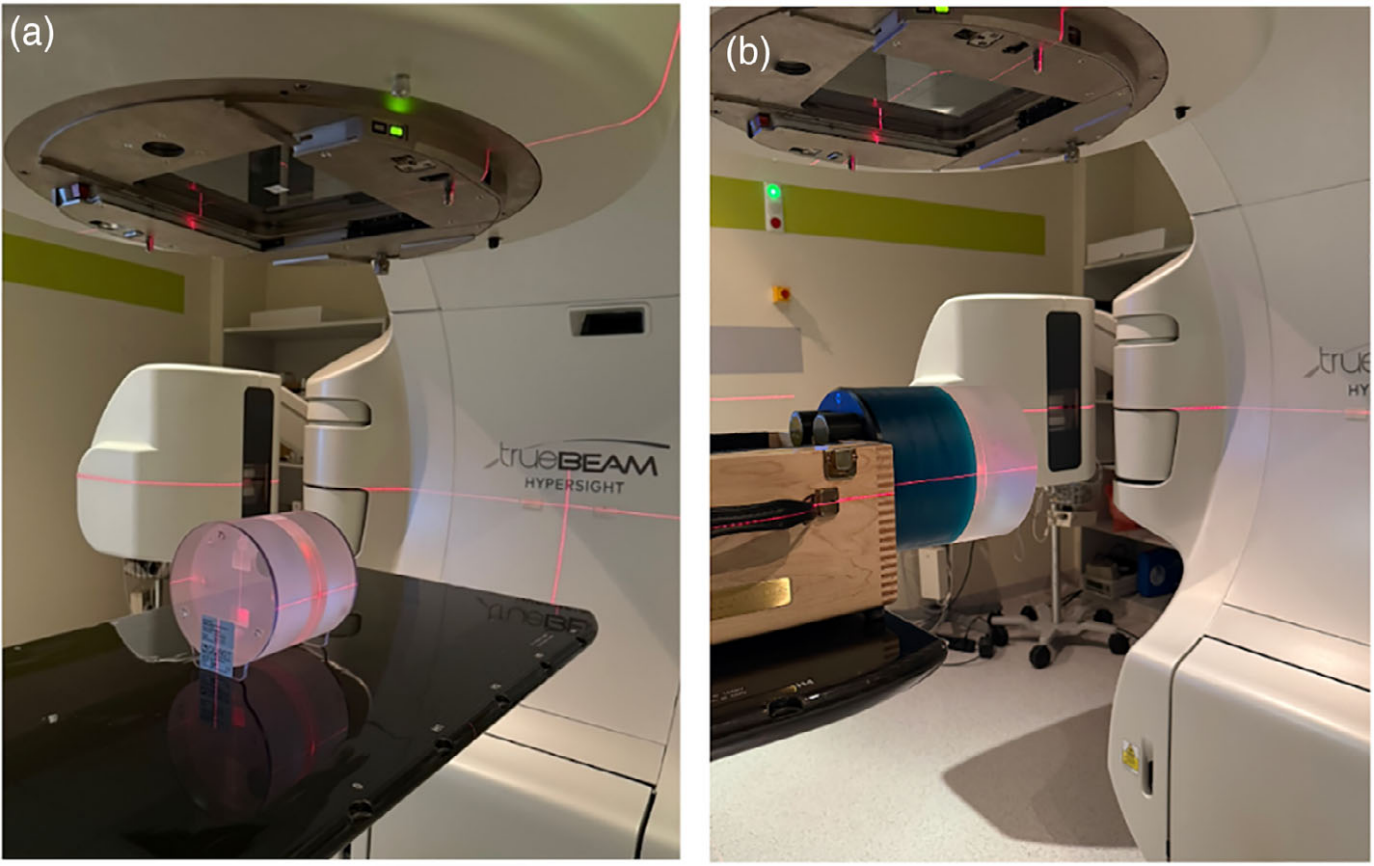
Measurement setup during kV‐CBCT acquisition: (a) QUART phantom positioned on the treatment couch, and (b) Catphan phantom mounted using a dedicated holder. Both phantoms were scanned using the same kV‐CBCT protocols and reconstruction settings, but with different positioning setups.

A cylindrical acrylic phantom with embedded resolution and noise rods (Figure 1a). We used MaximQA software to analyze kV‐CBCT images. Regions of interest (ROIs) were defined per the software defaults: for example, a 25 mm‐diameter circle in the uniform section for noise analysis, and a line‐pair gauge analysis for spatial resolution.^6^, ^7^

#### 2.2.2 Catphan 604 phantom

A modular phantom with low‐contrast spheres, high‐contrast resolution targets, and material inserts (Figure 1b). ARTISCAN software was used. ROIs for CNR and SNR were specified manually, for example, a 50 mm‐diameter circle for the background and small ROIs inside each insert. The software's “uniformity” metric was used similarly. The low‐contrast module yielded contrast‐detail detection diameters. Spatial resolution was estimated from the smallest visible line pair (via human visual check, with inter‐observer verification).^8^, ^9^

### 2.3 Metrics and data acquisition

Acquisitions were performed periodically at a frequency of three sessions per week, yielding a total of 9 measurement sessions. In each session, data were acquired for a set of clinically used kV‐CBCT protocols. Within each session, both phantoms were scanned using the same clinical kV‐CBCT protocols and reconstruction settings. Because QUART was positioned on the couch and Catphan on a dedicated holder, results were interpreted mainly in terms of stability and trends rather than direct equivalence of absolute values (Figure 1). For each acquisition, a set of image quality parameters was recorded, including contrast‐to‐noise metrics (CNR, SNR), image noise, uniformity, HU value stability for reference inserts, and in‐plane geometric reconstruction parameters.

Noise was taken as the standard deviation of HU in a uniform water‐equivalent region. SNR was defined as the signal (mean HU of a high‐contrast insert) divided by the noise (SD of the background). CNR was calculated as the absolute difference between the mean HU value of the insert and the mean HU value of the background, divided by the standard deviation of the background signal. HU uniformity was defined as the range of HU values across central and peripheral ROIs. Geometric accuracy was determined by measuring known distances between inserts that were 50 and 105 mm, respectively, for Catphan and QUART phantoms.^1^, ^3^, ^8^

In the comparative analysis of HU values for materials common to both phantoms, the following inserts were included: Acrylic, Air, Polystyrene, Teflon, and a water‐equivalent material (Water in the QUART phantom / Solid Water in the Catphan phantom). In addition, inserts present only in the Catphan phantom (e.g., LDPE, PMP, Delrin) were analyzed descriptively as a supplement to the characterization of HU stability over a broader range of radiation attenuation, without direct comparison with the QUART phantom (Table S1).

### 2.4 Statistical analysis

All comparisons between QUART/MaximQA and Catphan/ARTISCAN were paired by date and protocol. Data are summarized as mean ± SD and the coefficient of variation (CV), that is, a normalized measure of dispersion defined as the standard deviation relative to the mean. Lower CVs were interpreted as indicating greater stability and repeatability of results over time, whereas higher CV values indicated greater variability between successive imaging points. We used paired t‐tests when normality (Shapiro‐Wilk) was met; otherwise, Wilcoxon signed‐rank tests to compare each metric across phantoms. All tests were performed at the *p* < 0.01 significance level.

## 3 RESULTS

### 3.1 Noise, SNR and uniformity

Table 2 compares the descriptive statistics obtained for these parameters using QUART/MaximQA and Catphan/ARTISCAN measurement sets.

**TABLE 2:** Summary of descriptive statistics for the noise signal‐to‐noise (SNR) ratio and uniformity parameters, for selected IGRT protocols, received as a result of image analysis performed with QUART/MaximQA and Catphan/ARTISCAN sets.

		Mean + SD and (Coefficient of Variation [%])		
Parameter	Protocol	QUART/MaximQA	Catphan/ARTISCAN	*p*–value
Noise [HU]	Image Gently	31.0 + 1.2 (3.7)	54.0 + 8.3 (15.4)	< 0.01
	Head	14.5 + 0.4 (2.7)	27.8 + 1.4 (4.9)	< 0.01
	Thorax	9.9 + 0.4 (3.8)	9.1 + 0.2 (2.5)	< 0.01
	Pelvis	2.9 + 0.3 (9.3)	4.8 + 0.8 (15.9)	< 0.01
	Pelvis Large	2.6 + 0.2 (9.6)	3.7 + 0.4 (11.3)	< 0.01
SNR	Image Gently	35.2 + 1.2 (3.4)	19.3 + 1.9 (9.7)	< 0.01
	Head	76.4 + 2.1 (2.8)	36.5 + 3.9 (10.6)	< 0.01
	**Thorax**	**112.7 + 4.4 (3.9)**	**117.0 + 3.2 (2.7)**	**0.06**
	Pelvis	381.2 + 28.2 (9.3)	213.7 + 19.8 (9.2)	< 0.01
	Pelvis Large	495.1 + 40.7 (8.2)	283.6 + 31.5 (11.1)	< 0.01
Uniformity [HU]	**Image Gently**	**11.7 + 2.9 (25.0)**	**7.3 + 5.2 (70.3)**	**0.10**
	**Head**	**7.1 + 2.6 (36.2)**	**5.3 + 1.6 (29.4)**	**0.06**
	Thorax	16.1 + 1.5 (9.0)	12.0 + 3.3 (27.8)	< 0.01
	**Pelvis**	**13.2 + 2.0 (15.0)**	**10.2 + 4.4 (43.3)**	**0.05**
	Pelvis Large	14.9 + 1.3 (8.5)	7.6 + 2.8 (36.5)	< 0.01

Statistical comparison performed at *a* = 0.01. The observations with no statistically significant differences are bolded.

In general, higher, statistically significant noise was detected in the Catphan/ARTISCAN set, except in the Thorax protocol, where higher QUART/MaximQA values were observed. Higher SNR values were observed for QUART/MaximQA in most protocols; however, these differences should be interpreted in the context of system‐specific ROI definitions and analysis procedures. The exception was the Thorax protocol, for which the results did not differ statistically. The uniformity was comparable across the Image Gently and Head and Pelvis protocols. For the Thorax and Pelvis Large protocols, the coefficients of variation were higher for measurements performed with Catphan/ARTISCAN, indicating higher variability of this parameter in the Catphan/ARTISCAN measurements for these protocols.

### 3.2 Geometry

Geometric accuracy was assessed by evaluating geometric distortion in the horizontal and vertical directions. Due to different reference distances of 50 and 105 mm for the Catphan and QUART phantoms, respectively, the ratio of measured to reference distance (RD = (measured distance) / (reference distance) * 100 [%]) and CV were considered during analysis (Table 3).

**TABLE 3:** Averaged ratios of measured to reference distance (RD [%]) and coefficient of variation (CV [%]), for selected IGRT protocols, received as a result of image analysis in horizontal and vertical directions, and performed with QUART/MaximQA and Catphan/ARTISCAN sets.

	Ratio of measured to reference distance [%] and (Coefficient of Variation [%])		
Protocol	QUART/MaximQA	Catphan/ARTISCAN	*p* – value
Horizontal direction			
Image Gently	99.9 (0.1)	99.9 (0.2)	>> 0.01
Head	99.9 (0.1)	100.0 (0.3)	>> 0.01
Thorax	99.9 (0.1)	99.8 (0.4)	>> 0.01
Pelvis	99.9 (0.1)	99.6 (0.5)	>> 0.01
Pelvis Large	99.9 (0.1)	99.6 (0.2)	>> 0.01
Vertical direction			
Image Gently	100.0 (0.1)	99.9 (0.2)	>> 0.01
Head	99.9 (0.1)	100.0 (0.3)	>> 0.01
Thorax	100.0 (0.1)	99.8 (0.4)	>> 0.01
Pelvis	100.0 (0.1)	99.6 (0.5)	>> 0.01
Pelvis Large	100.0 (0.1)	99.6 (0.2)	>> 0.01

Statistical comparison performed at *a* = 0.01.

In both phantoms, the metrics (RD and CV) analyzed along horizontal and vertical direction remained stable across consecutive acquisitions, indicating the absence of significant geometric deviations in the analyzed protocols.

### 3.3 The stability of HU across different insert materials

Table 4 compares the HUs obtained for Acrylic, Air, Polystyrene, Teflon, and a water‐equivalent material inserts using QUART/MaximQA and Catphan/ARTISCAN measurement sets. The HUs obtained for the Catphan‐specific inserts are listed in the supplementary materials.

**TABLE 4:** HU values for material inserts (Acrylic, Air, Polystyrene, Teflon, and a water‐equivalent material: Water in the QUART phantom/solid water in the Catphan phantom), for selected IGRT protocols, received as a result of image analysis performed with QUART/MaximQA and Catphan/ARTISCAN sets. Statistical comparison performed at a = 0.01. The observations with no statistically significant differences are bolded.

		Mean + SD [HU] and (Coefficient of Variation [%])		
Insert	Protocol	QUART/MaximQA	Catphan/ARTISCAN	*p* – value
Acrylic	Image Gently	82.8 + 5.0 (6.0)	104.9 + 5.0 (4.7)	< 0.01
	Head	97.1 + 2.8 (2.9)	113.5 + 2.2 (1.9)	< 0.01
	Thorax	124.7 + 1.9 (1.5)	128.7 + 1.6 (1.2)	< 0.01
	Pelvis	121.4 + 2.2 (1.9)	126.8 + 2.2 (1.7)	< 0.01
	**Pelvis Large**	**126.8 + 2.1 (1.7)**	**128.5 + 1.0 (1.2)**	**0.06**
Air	Image Gently	−998.2 + 0.8 (−0.1)	−985.5 + 4.0 (0.4)	< 0.01
	Head	−999.4 + 0.5 (−0.1)	−987.6 + 3.2 (0.3)	< 0.01
	Thorax	−992.3 + 1.0 (−0.1)	−978.6 + 5.8 (0.6)	< 0.01
	Pelvis	−1000.0 + 0.1 (−0.1)	−980.5 + 6.6 (0.7)	< 0.01
	Pelvis Large	−997.0 + 0.5 (−0.1)	−977.1 + 7.5 (0.8)	< 0.01
Polystyrene	Image Gently	−59.6 + 2.1 (−3.6)	−65.3 + 4.3 (−6.6)	< 0.01
	**Head**	−**44.4 + 2.1 (**−**4.7)**	−**48.8 + 2.9 (**−**6.0)**	**0.01**
	Thorax	−19.3 + 1.5 (−7.8)	−31.8 + 1.4 (−4.4)	< 0.01
	Pelvis	−25.9 + 2.3 (−8.7)	−32.8 + 1.2 (−3.6)	< 0.01
	Pelvis Large	−19.3 + 1.9 (−10.0)	−29.1 + 1.3 (−4.4)	< 0.01
Teflon	**Image Gently**	**985.8 + 5.7 (0.6)**	**986.3 + 12.4 (1.3)**	**0.92**
	**Head**	**967.9 + 8.8 (0.9)**	**956.6 + 5.1 (0.5)**	**0.02**
	Thorax	953.8 + 2.8 (0.3)	940.2 + 4.5 (0.5)	< 0.01
	Pelvis	973.6 + 2.5 (0.3)	939.7 + 14.1 (1.5)	< 0.01
	Pelvis Large	963.4 + 3.6 (0.4)	928.5 + 5.0 (0.5)	< 0.01
Water‐equivalent^*^ QUART: Water, Catphan: Solid Water	Image Gently	−3.0 + 3.0 (−100.0)	35.5 + 5.4 (15.3)	< 0.01
	Head	−7.6 + 1.9 (−25.7)	47.8 + 4.8 (10.0)	< 0.01
	Thorax	3.8 + 1.6 (41.4)	61.6 + 1.6 (2.7)	< 0.01
	Pelvis	1.8 + 2.6 (148.2)	60.3 + 3.1 (5.1)	< 0.01
	Pelvis Large	4.8 + 2.1 (42.9)	59.5 + 2.0 (3.3)	< 0.01

*CV for materials with mean HU close to 0 (in particular, the water in the QUART phantom) should be interpreted with caution, because under such conditions, CV is unstable and strongly dependent on the mean value. The observed differences in absolute HU between the water (QUART) and solid water (Catphan) result from differences in the reference materials and their definitions in the analytical systems; therefore, these results were interpreted as a comparison of stability within a given phantom and protocol, rather than as a direct comparison of nominal agreement with water.

**FIGURE 2:**
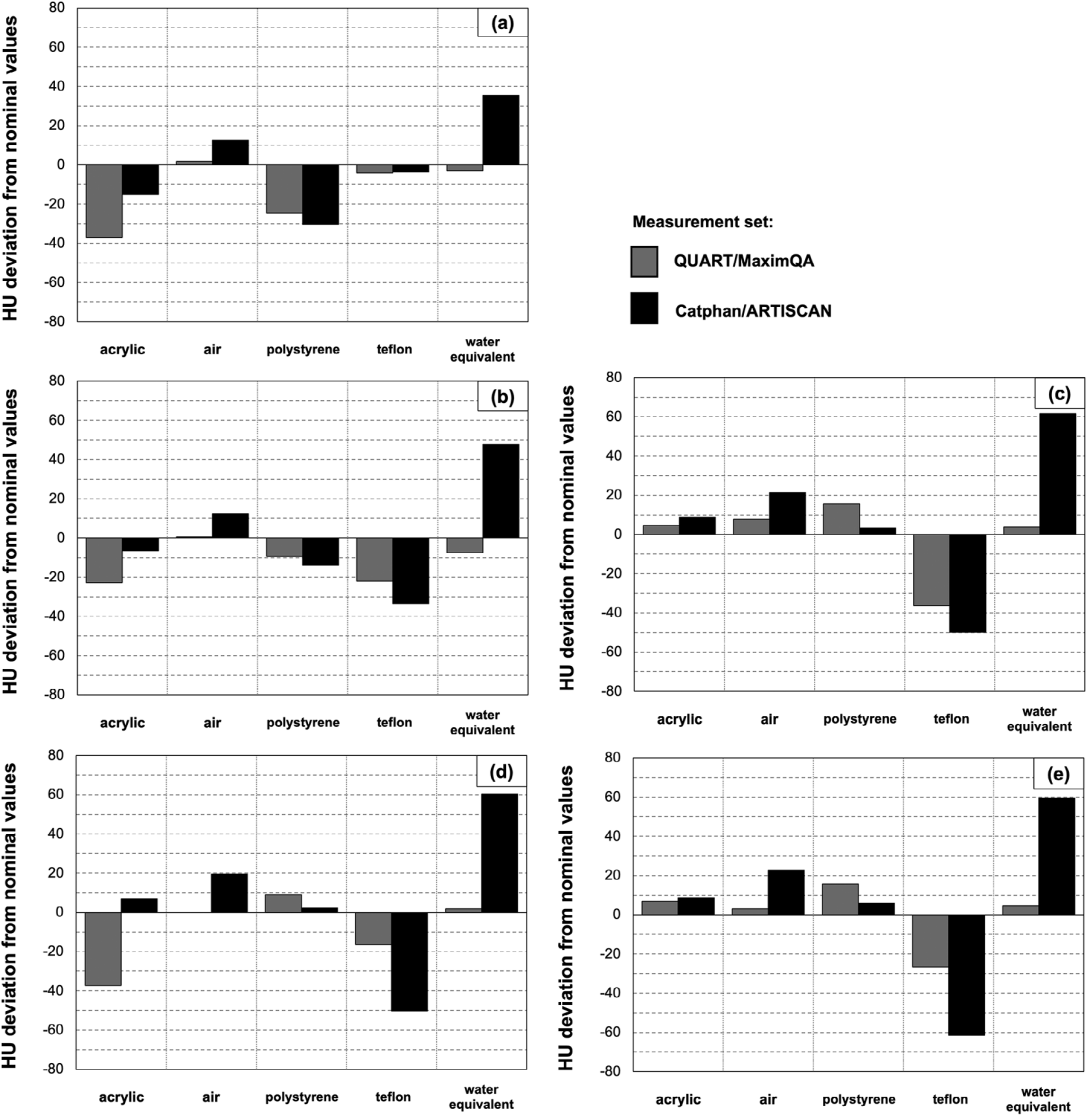
Deviations of HU related to nominal values, respectively for acrylic, air, polystyrene, teflon, and a water‐equivalent material (QUART: water, Catphan: solid water) of the inserts imaged through (a) image gently, (b) head, (c) thorax, (d) pelvis, and (e) pelvis large protocols. Results for QUART/MaximQA (grey bar‐chart) and Catphan/ARTISCAN (black bar‐chart) sets. Nominal HU values are: 120 HU for acrylic, −1000 HU for air, −35 HU for polystyrene, 990 HU for teflon and 0 HU for water‐equivalent insert.

Additionally, deviations from nominal HU (i.e., 120 HU for acrylic, −1000 HU for air, −35 HU for polystyrene, 990 HU for teflon and 0 HU for water‐equivalent insert) were calculated for results obtained from both measurement sets (Figure 2 and Table S2, included in the supplementary data).

The results presented in Table 4 and Figure 2 demonstrate differences in HU behavior between the QUART/MaximQA and Catphan/ARTISCAN systems, reflecting phantom‐specific material properties, kVp dependence and analysis methodology.

### 3.4 CNR and spatial resolution

Because QUART/MaximQA reports contrast performance using CNR rather than dedicated resolution targets, CNR was reported for QUART, whereas spatial resolution was assessed with Catphan/ARTISCAN. Accordingly, Table 5 presents QUART CNR values separately from Catphan high‐contrast resolution [lp/cm] and low‐contrast detectability. These parameters were not treated as directly equivalent metrics, because the phantoms contain different test modules and rely on different analysis procedures. They were therefore interpreted as complementary QA indicators rather than as direct head‐to‐head measurements.

**TABLE 5:** The Contrast‐to‐Noise Ratio (CNR) [%] acquired on the QUART phantom, and the high‐contrast [lp/cm] and low‐contrast [%] resolutions gathered on the Catphan phantom.

	QUART/MaximQA	Catphan/ARTISCAN	
		Resolution	
	Contrast‐to‐Noise Ratio [%]	High‐Contrast [lp/cm]	Low‐Contrast [number of visible disks]
Protocol	*Mean + SD (Coefficient of Variation [%])*		
Image gently	5.9 + 0.4 (6.0)	7.0 + 0.5 (6.7)	0
Head	12.1 + 1.0 (8.5)	7.2 + 0.3 (4.1)	0
Thorax	12.5 + 0.8 (6.4)	5.4 + 0.3 (4.9)	5 (1%^*^)
Pelvis	38.8 + 5.2 (15.1)	5.2 + 0.2 (4.2)	4 (1%^*^)
Pelvis large	41.8 + 3.1 (7.5)	5.4 + 0.2 (4.4)	4 (0.5%^*^)

*Values in parentheses indicate the nominal contrast level of the low‐contrast disks in the Catphan module (relative to the background); the Low‐Contrast column reports the number of disks scored as visible at the corresponding contrast level.

## 4 DISCUSSION

In our study, noise was significantly lower and SNR significantly higher with QUART/MaximQA compared to Catphan/ARTISCAN in nearly all protocols (e.g., Pelvis Large SNR 495.1 vs 283.6, *p* < 0.01).^7^ However, these differences should be interpreted with caution, as they may be influenced by phantom geometry, couch contribution and software‐specific analysis methods. These differences can affect QA thresholds: QA phantoms producing different absolute values should still use baseline comparisons.^2^, ^10^

### 4.1 Uniformity

Both systems showed small uniformity variations (< 5 HU) in central slices, generally not statistically significant. This agrees with reports that uniformity is a sensitive CBCT QA metric (it was the most predictive in Manger et al. 2019).^10^ For example, the largest uniformity deviation observed was 16.1 HU (Thorax, QUART) vs 12.0 HU (Thorax, Catphan) with *p* < 0.01. However, these differences are within TG‐142 tolerances (± 40 HU),^2^ so both systems meet QA criteria.

### 4.2 Geometric accuracy

Both phantoms yielded nearly ideal geometric fidelity. Measured distances were ∼99.6%–100.0% of the nominal value in both the horizontal and vertical directions across all protocols (Table 3), with no significant differences. This shows that both QA systems provide excellent spatial calibration for CBCT. These results satisfy the AAPM TG‐142‐recommended tolerances (< 2 mm) and corroborate other studies reporting geometry errors < 1 mm.^2^, ^7^ Thus, either phantom can reliably assess CBCT geometry.

### 4.3 HU constancy

HU values differed between systems, reflecting phantom‐specific material properties, beam‐quality dependence and analysis methodology. This interpretation is consistent with Peng et al. (2021),^7^ who found QUART HUs within ± 50 HU of nominal. These HU differences underscore the need for baseline HU references to be adjusted for phantom‐specific effects in QA.

### 4.4 Contrast and resolution

Contrast and resolution were assessed with different phantom‐specific endpoints. QUART/MaximQA provided CNR values, whereas Catphan/ARTISCAN provided high‐contrast spatial resolution and low‐contrast detectability. These readouts were not analyzed as directly equivalent metrics, because the two systems use different test objects, ROI definitions, and analysis workflows. They should therefore be interpreted as complementary indicators of image‐quality stability within routine QA rather than as interchangeable quantitative measures. Across protocols, QUART CNR ranged from 5.9 to 41.8%, whereas Catphan high‐contrast spatial resolution ranged from 5.2 to 7.2 lp/cm. These findings align with the literature, which notes the lack of dedicated low‐contrast features in QUART and the reliance on alternative metrics.^7^

In general, the results support and extend previous kV‐CBCT QA findings. Like Taneja et al. (2020), we confirm that kV‐CBCT image quality metrics vary by protocol and phantom, emphasizing the need for technique‐specific baselines.^11^ Our observation of higher SNR with QUART is consistent with its design (transparent, low‐scatter phantom) compared to the denser Catphan. Manger et al. (2019) highlighted uniformity as a key metric^10^; our data similarly show stable uniformity in routine QA. Peng et al. (2021) using a Halcyon CBCT with QUART also reported HU accuracy within ± 50 HU and variable SNR/CNR by protocol,^7^ in agreement with our findings under standard protocols.

Overall, the results should be interpreted as a comparison of two clinically implemented QA procedures rather than a strict physical equivalence test of two phantoms. The observed differences in absolute values may reflect not only image quality, but also phantom geometry, material composition, ROI definition, couch contribution and software‐specific processing.

### 4.5 Limitations

This work focused on phantom QA metrics rather than clinical images. No patient data or ethical approvals were required. The study did not evaluate dose, artefacts, or application to adaptive planning. The QUART lacks resolution targets, so we relied on the Catphan for spatial measurements, which were visually assessed and should be interpreted as supportive QA indicators. All data are from one linac model; data from other machines, including multi‐institutional machines, might differ. However, our comprehensive testing across multiple clinical protocols improves generalizability. Nevertheless, several methodological factors should be considered when interpreting the results. The QUART and Catphan phantoms differ in geometry, material composition and available test modules, which prevents complete one‐to‐one comparison of all image‐quality metrics. ROI definition and placement were software‐specific and may have influenced noise, SNR and CNR values. The Catphan Solid Water insert was not considered equivalent to liquid water at 0 HU; therefore, HU‐related results were interpreted mainly as stability metrics. HU values may also depend on beam quality, and the use of fixed nominal values across protocols ranging from 80 to 140 kV may influence apparent HU deviations, especially for Teflon and Polystyrene. The study was limited to nine measurement sessions per protocol, and multiple statistical comparisons were performed without formal correction for multiple testing.

## 5 CONCLUSIONS

QUART/MaximQA and Catphan/ARTISCAN both yield consistent kV‐CBCT QA metrics that satisfy AAPM TG‐142/TG‐179 recommendations. QUART/MaximQA showed significantly lower measured noise and higher measured SNR across most protocols, while HU differences should be interpreted in relation to phantom‐specific material properties (e.g., true water vs Catphan solid water). Geometric distortions were negligible for both phantoms. We recommend using phantom‐ and workflow‐specific baselines, including a local baseline for the Catphan Solid Water insert rather than assuming equivalence to 0 HU. In practice, either system can monitor kV‐CBCT performance, but absolute QA values should not be considered directly interchangeable between phantom/software systems.

## AUTHOR CONTRIBUTIONS

Karolina Sanocka, Bartosz Pawałowski and Urszula Sobocka‐Kurdyk conceived and designed the study. Karolina Sanocka, Ewelina Nowak, Zuzanna Wróblewicz, Maksymilian Wosicki and Sebastian Gajny performed the experiments and acquired the data. Bartosz Pawałowski, Krzysztof Matuszewski and Tomasz Piotrowski analyzed and interpreted the data. Karolina Sanocka, Olga Jankowiak drafted the manuscript. Bartosz Pawałowski and Tomasz Piotrowski critically revised the manuscript for important intellectual content. All authors read and approved the final manuscript.

## CONFLICT OF INTEREST STATEMENT

The authors declare no conflicts of interest.

## ETHICAL APPROVAL

The authors declare that this study did not involve human participants or animals.

## Supporting information




**Supporting Information: Table S1**. HU values for material Catphan‐specific inserts (Delrin, PMP, LDPE), for selected IGRT protocols, received as a result of image analysis using ARTISCAN software.

**Supporting Information: Table S2**. Deviations of HU related to nominal values (ΔHU), respectively for acrylic, air, polystyrene, teflon, and a water‐equivalent material (QUART: water, Catphan: solid water) of the inserts imaged through (a) image gently, (b) head, (c) thorax, (d) pelvis, and (e) pelvis large protocols.

## Data Availability

The datasets generated during and/or analyzed during the current study are available from the corresponding author on reasonable request.
