# Supplementary material for: Routine kV‐CBCT quality assurance in IGRT: Workflow‐based comparison of QUART/MaximQA vs Catphan/ARTISCAN
Source: J Appl Clin Med Phys. 2026 Jul 15;27(7):e70708. doi: 10.1002/acm2.70708 (PMC13373441; doi:10.1002/acm2.70708)
Supplement: Supplementary file 1 — Supporting Information: Table S1. HU values for material Catphan‐specific inserts (Delrin, PMP, LDPE), for selected IGRT protocols, received as a result of image analysis using ARTISCAN software. [file ACM2-27-e70708-s002.docx]

**Table S-1.** HU values for material Catphan -specific inserts (Delrin, PMP, LDPE), for selected IGRT protocols, received as a result of image analysis using ARTISCAN software.

| Insert | Protocol | Mean + SD [HU] and  (Coefficient of Variation [%]) |
| --- | --- | --- |
| Delrin | Image gently | 340.4 + 10.4 (3.1) |
|  | Head | 341.6 + 2.3 (0.7) |
|  | Thorax | 356.6 + 2.6 (0.7) |
|  | Pelvis | 356.1 + 5.7 (1.6) |
|  | Pelvis large | 350.9 + 2.2 (0.6) |
| PMP | Image gently | -213.4 + 4.6 (-2.2) |
|  | Head | -193.3 + 1.7 (-0.9) |
|  | Thorax | -178.5 + 2.0 (-1.1) |
|  | Pelvis | -178.5 + 3.9 (-2.2) |
|  | Pelvis large | -172.8 + 2.0 (-1.5) |
| LDPE | Image gently | -120.5 + 6.2 (-5.1) |
|  | Head | -105.5 + 3.0 (-2.8) |
|  | Thorax | -88.4 + 1.6 (-1.8) |
|  | Pelvis | -89.2 + 2.4 (-2.6) |
|  | Pelvis large | -85.3 + 2.9 (-3.9) |
