# Supplementary material for: Routine kV‐CBCT quality assurance in IGRT: Workflow‐based comparison of QUART/MaximQA vs Catphan/ARTISCAN
Source: J Appl Clin Med Phys. 2026 Jul 15;27(7):e70708. doi: 10.1002/acm2.70708 (PMC13373441; doi:10.1002/acm2.70708)
Supplement: Supplementary file 2 — Supporting Information: Table S2. Deviations of HU related to nominal values (ΔHU), respectively for acrylic, air, polystyrene, teflon, and a water‐equivalent material (QUART: water, Catphan: solid water) of the inserts imaged through (a) image gently, (b) head, (c) thorax, (d) pelvis, and (e) pelvis large protocols. [file ACM2-27-e70708-s001.docx]

**Table S-2.** Deviations of HU related to nominal values (ΔHU), respectively for acrylic, air, polystyrene, teflon, and a water-equivalent material (QUART: water, Catphan: solid water) of the inserts imaged through (a) image gently, (b) head, (c) thorax, (d) pelvis, and (e) pelvis large protocols.

| Insert | Protocol | Nominal HU | QUART/MaximQA | Catphan/ARTISCAN |
| --- | --- | --- | --- | --- |
|  |  |  | HU deviations related to nominal values [HU] | |
| Acrylic | Image gently | 120 | -37.22 | -15.11 |
|  | Head |  | -22.89 | -6.49 |
|  | Thorax |  | 4.67 | 8.66 |
|  | Pelvis |  | -37.22 | 6.83 |
|  | Pelvis large |  | 6.78 | 8.54 |
| Air | Image gently | -1000 | 1.78 | 12.52 |
|  | Head |  | 0.56 | 12.42 |
|  | Thorax |  | 7.67 | 21.25 |
|  | Pelvis |  | 0.00 | 19.47 |
|  | Pelvis large |  | 3.00 | 22.89 |
| Polystyrene | Image gently | -35 | -24.56 | -30.27 |
|  | Head |  | -9.44 | -13.84 |
|  | Thorax |  | 15.67 | 3.17 |
|  | Pelvis |  | 9.11 | 2.25 |
|  | Pelvis large |  | 15.67 | 5.91 |
| Teflon | Image gently | 990 | -4.22 | -3.67 |
|  | Head |  | -22.11 | -33.44 |
|  | Thorax |  | -36.22 | -49.83 |
|  | Pelvis |  | -16.44 | -50.33 |
|  | Pelvis large |  | -26.56 | -61.55 |
| Water - equivalent | Image gently | 0 | -3.00 | 35.47 |
|  | Head |  | -7.56 | 47.83 |
|  | Thorax |  | 3.78 | 61.59 |
|  | Pelvis |  | 1.78 | 60.33 |
|  | Pelvis large |  | 4.78 | 59.51 |
